# Rapid Recognition of Field-Grown Wheat Spikes Based on a Superpixel Segmentation Algorithm Using Digital Images

**DOI:** 10.3389/fpls.2020.00259

**Published:** 2020-03-06

**Authors:** Changwei Tan, Pengpeng Zhang, Yongjiang Zhang, Xinxing Zhou, Zhixiang Wang, Ying Du, Wei Mao, Wenxi Li, Dunliang Wang, Wenshan Guo

**Affiliations:** ^1^Jiangsu Key Laboratory of Crop Genetics and Physiology/Jiangsu Co-Innovation Center for Modern Production Technology of Grain Crops/Joint International Research Laboratory of Agriculture and Agri-Product Safety of the Ministry of Education of China, Yangzhou University, Yangzhou, China; ^2^College of Agronomy, Hebei Agricultural University/Key Laboratory of Crop Growth Regulation of Hebei Province, Baoding, China; ^3^Station of Land Protection of Yangzhou City, Yangzhou, China

**Keywords:** wheat spike, recognition, superpixel segmentation, digital images, estimation

## Abstract

Wheat spike number, which could be rapidly and accurately estimated by the image processing technology, serves as the basis for crop growth monitoring and yield prediction. In this research, simple linear iterative clustering (SLIC) was performed for superpixel segmentation of the digital images of field-grown wheat. Firstly, certain characteristic color parameters were extracted and analyzed from the digital images, and the classifiers with the highest accuracy were chosen for subsequent image classification. Next, the main body of wheat spike was extracted through a series of morphological transformation and estimate was performed for each region. Backbone of the head was extracted, and the number of inflection points of backbone was detected. Then the wheat spike number was determined by combining the estimate of inflection points of backbone and the estimate for each region. Finally, the wheat spike number estimate was verified under four nitrogen fertilizer levels. The results were as follows: (1) Super green value (Eg) and normalized red green index (Dgr) were used as classification features to recognize wheat spikes, soil and leaves; (2) compared with pixel-based image processing, wheat spike recognition effect was much better after superpixel segmentation, as the main body of wheat spike extracted was more clear and morphology more intact; and (3) wheat plants had better growth under high nitrogen fertilizer level, and the accuracy of wheat spike number estimation was also the highest, which was 94.01%. The growth status was the worst under no nitrogen fertilizer application, and the accuracy of wheat spikes number estimation was also the lowest, which was only 80.8%. After excluding the no nitrogen condition, the accuracy of wheat spikes number estimation among mixed samples with more uniform growth status was up to 93.8%, which was an increase by 10.1% than before the exclusion. Wheat spikes number estimate based on superpixel segmentation and color features was a rapid and accurate method that was applicable to the field environment. However, this method was not recommended for use when the growth status of wheat was poor or of high heterogeneity. The findings provided reference for field-grown wheat yield estimate.

## Introduction

Wheat has been the most important cereal crop worldwide and also one of the most important food crops in China. For China, a major importer of wheat, standardization of production management and quality stabilization of wheat varieties were key pathways toward yield improvement, and this in turn was closely related to the national economy and food security. Number of spikes per unit area has been an important component of wheat yield, and fast and accurate estimate of wheat spike number was of high significance for high-yield cultivation and superior species selection and breeding (Nerson, 1980; Siddique and Whan, 1993). However, conventional field survey based on manpower was time- and labor-consuming. Along with the rising of agricultural informatization and mechanization level, image processing technology has found extensive applications in crop production. Moreover, computer vision with its advantages of high precision and intelligence attracted it as an alternative to human inspection. This technology was a dramatic boost for pest detection (Boissard et al., 2008; Shahin and Symons, 2011; Ding and Taylor, 2016; Senthilkumar et al., 2017), growth monitoring (Clevers and Leeuwen, 1996; Chaerle and Straeten, 2000; Wang et al., 2013; Silva et al., 2014), yield prediction (Salazar et al., 2007; Dunn and Martin, 2010; Aggelopoulou et al., 2011; Aguate et al., 2017) and species recognition (Neuman et al., 1987; Lópezgranados et al., 2006; Tellaeche et al., 2011; Pantazi et al., 2016).

A large number of studies have been conducted on capturing wheat phenotypic traits by using the image processing technology. Jin et al. (2017) proposed a method for high-throughput phenotype information extraction of wheat seedling density in field environment during the seedling stage by using images from low-altitude high-resolution unmanned aerial vehicle. Hosoi and Omasa (2009) used three-dimensional portable lidar imaging technology to estimate the density profile of vertical planting area and growth parameters of wheat canopy at different growth stages, and achieved good results. Walter et al. (2017) performed 3D point cloud processing to estimate wheat canopy height and harvest index reliably. The model reliability was further improved by increasing the number of images fitted. Wheat spike was one of the important agronomic components, and accurate determination of wheat spike number was very important for estimating wheat yield, which was the key step of field phenotype study (Zhang et al., 2007). spikeDue to the complexity of the field environment (e.g. light intensity, soil reflectivity, weeds, etc. changing color, texture and shape of the image of wheat spike), accurate segmentation and identification of wheat spike has remained a major challenge. Balasubramaniam et al. proposed intuitionistic fuzzy C-means color clustering algorithm to segment the nutrition-deficient pixels in crop images after normalization. By comparing other methods, the effectiveness of this method was proved (Balasubramaniam and Ananthi, 2016). Li et al. (2017) detected wheat spikes by using the neural network based on Laws texture energy measure. The detection effect was improved by combining area and height thresholds to well over 80%. The spike area was effectively measured as well (Li et al., 2017). Schirrmann et al. (2016) used the wheat field images obtained by unmanned aerial vehicle (UAV) to analyze the relationship between biophysical parameters and image variables, proving the applicability of UAV images in identifying the temporal and spatial patterns of wheat canopy development. Zhou et al. (2018) proposed a new algorithm that used computer vision to accurately identify wheat spikes in digital images, and adopted multi-feature optimization and a twin-support-vector-machine segmentation (TWSVM-Seg) model to determine the number of spikes. Jose et al. applied Laplacian filter and median filter to the digital wheat photos captured in field environment to extract the main part of wheat spikes. Peak detection algorithm was used to extract image peaks and to determine spike number. Moreover, the relationship between spike number and yield at different stages was analyzed, and it was found that the spike number at the flowering stage had the highest correlation to yield (Fernandez-Gallego et al., 2018).

Previous studies on wheat spike recognition were mostly based on pixel segmentation, and the influence of varying growth status on the recognition effect was rarely considered. Here, after certain pre-processing, pixels on the digital images of wheat were grouped together into superpixels, and wheat spikes were recognized based on superpixel segmentation, to reduce the interference from non-relevant pixels in the process of extracting image features and to improve the recognition effect. Moreover, variation of wheat growth status was simulated by applying nitrogen gradient to the seedlings, and the wheat spike number estimate was compared under different nitrogen fertilizer application levels, to improve the reliability of wheat spike number estimate. In order to estimate wheat spike number in field environment rapidly and accurately, simple linear iterative clustering (SLIC) was applied to the digital images for superpixel segmentation, and the wheat spikes were recognized based on color features. Then backbone of the head was extracted and the wheat spike number was estimated based on the number of inflection points of backbone. Accuracy of wheat spike number estimate was compared under different nitrogen fertilizer application levels. The influence of varying growth status on the recognition effect was discussed. The purpose was to provide a new reliable pathway to accurate wheat spike estimate.

## Materials and Methods

### Experimental Design

**FIGURE 1:**
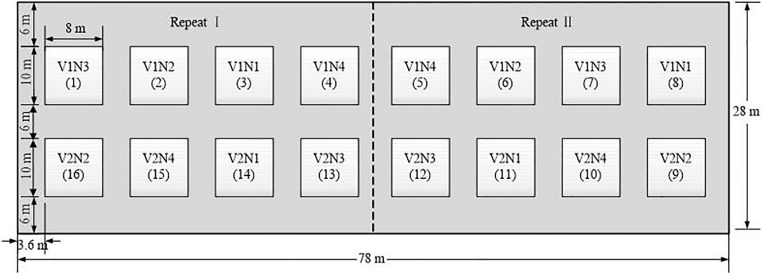
Experimental design. (V1 and V2 represent *Yangmai 16* and *Yangmai 17* varieties, respectively. N1, N2, N3 and N4 represent different nitrogen treatments of 0, 225 kg⋅ha**^–^**^1^,450 kg⋅ha**^–^**^1^,900 kg⋅ha**^–^**^1^, respectively. Each level had two replicates. A total of 16 plots, the area of each experimental plot was 8*10 m^2^).

Experiments were conducted in the experimental field of Agricultural College of Yangzhou University in 2018 (119°23′26″ E, 32°23′53″ N), and a completely randomized design was adopted (Figure 1). Representative wheat cultivars were *Yangmai 16* and *Yangmai 17*, both were spring wheat cultivars with medium maturity and long awn, and the spike types were spinning type and rectangle type, respectively. The former crop was rice, while sandy loam was the soil texture. In the soil layer of 0–30 cm, soil organic matter was 22.7 g⋅kg^–1^ and available nitrogen was 101.8 mg; moreover, available phosphorus was 27.2 mg and available potassium was 84.6 mg. To investigate differences in the growth and biochemical composition of wheat, four levels of nitrogen fertilizer application (urea) were set up, namely, 0 (N1), 225 kg⋅ha^–1^ (N2, 1/2 of the normal level), 450 kg⋅ha^–1^ (N3, normal level), and 900 kg⋅ha^–1^ (N4, excessive level). Each level had two replicates, and 16 plots for the two winter wheat varieties were prepared. Other field management measures were administered as usual. Data source was digital images of wheat in the field.

### Data Acquisition

#### Acquisition of Digital Images of Wheat

At 5:00 p.m. on May 22st, 2018 (grain filling stage, solar zenith angle 65°04′54″, azimuth 94°38′27″), which was a sunny windless day, digital images of wheat were shot with SONY DSC-H9 camera against the light and in a vertical direction. The filming height was about 1m above the wheat canopy. The area shot was about 0.75 m^2^ per image. For each plot, four wheat images with resolution of 2592^∗^1944 were shot, thus 64 images were obtained in total.

#### Artificial Wheat Spike Number Estimate

**FIGURE 2:**
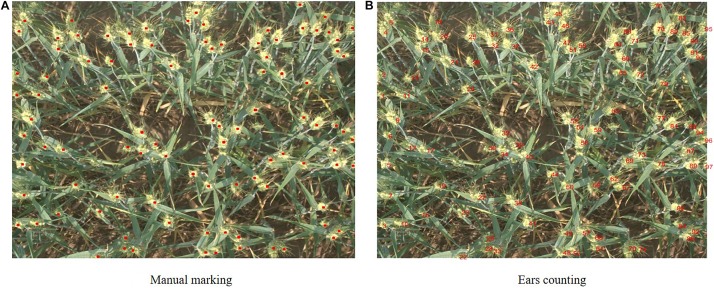
Wheat spikes manual counting process. [The location of wheat spikes were manually marked and the marked points were extracted and counted. The red points marked in panel **(A)** indicated the position of wheat spikes, and the red numbers in panel **(B)** indicated the serial number of wheat spikes in the picture].

Artificial and automatic wheat spike number estimate was combined in this study (Figure 2). First, the digital images of wheat were interpreted by researchers, who marked out the portions of wheat spikes. Then the marked points were extracted from the images using MATLAB R2016a. These points were counted and numbered in the images so as to accurately and intuitively determine the wheat spike number.

### Method of Wheat Spike Recognition

**FIGURE 3:**
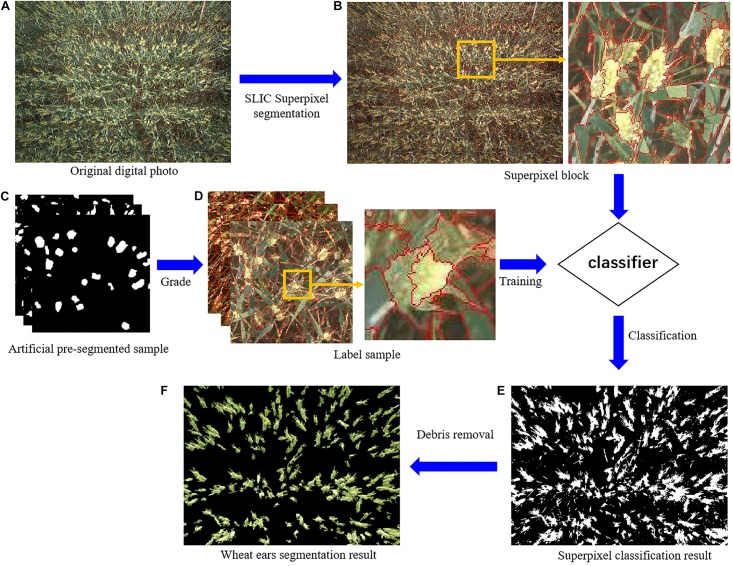
Wheat spikes recognition process. [**A** was the original digital photo. SLIC was Simple linear iterative clustering. **(B)** was the superpixel block. **(C)** was the artificial pre-segmentation sample. **(D)** was the label sample. Two classifiers were support vector machine (SVM) and K nearest neighbor (KNN). **(E)** was the superpixel classification result, and **F** was the wheat spikes segmentation result].

Wheat spike recognition consisted of the following steps: superpixel segmentation, sample labeling, color feature analysis, classifier training and recognition (Figure 3). To be specific,

(1) SLIC-based superpixel segmentation was applied to the digital images of wheat for pre-processing. Superpixels refer to the image blocks composing adjacent pixels with homogeneous features. Superpixel segmentation, which is to group these pixels into superpixels, has been widely used in image pre-processing (Kavzoglu and Tonbul, 2018). SLIC is the representative algorithm among a myriad of superpixel segmentation methods. SLIC is based on color similarity and spatial distance and employs K-means clustering for local iterative clustering to form superpixels (Radhakrishna et al., 2012; Akyilmaz and Leloglu, 2016; Zu et al., 2019). This algorithm involves two key parameters: pixel number of pre-segmetation (*n*) and pixel compactness (*m*). Pixel number *n* is the number of clusters centers in the image, which is uniformly assigned in the image according to the preset *n*. Generally speaking, the larger the *n*, the smaller the superpixels and the better the segmentation effect, though this may bring about the problems of higher calculation load and lower overall efficiency. Therefore, the *n* value should be reasonably set according to image size. Pixel compactness *m* is the weight assigned to the maximum distance within each cluster (including spatial distance and color distance). The larger the *m* value, the more regular the superpixel boundaries will be. For a more complex image, a smaller *m* value is usually needed. The main parameters of the algorithm included pixel number and pixel compactness of pre-segmentation, which were set to 10,000 and 10 based on the size of images used in the experiment (2592^∗^1944).
(2) Under each nitrogen fertilizer application level (regardless of wheat variety), five wheat images of 500^∗^500 were randomly clipped as samples for artificial pre-segmentation. Then the superpixels were labeled based on the results of artificial pre-segmentation. That is, pixels with proportion of wheat spikes exceeding 0.8 were labeled 1, and those below it 0.
(3) Some commonly used color indices were analyzed and appropriate ones were chosen as classification features (Stajnko et al., 2004; Moffett and Gorelick, 2013), which included super green value (E_*g*_), normalized red green index (D_*gr*_) and normalized blue green index (D_*gb*_), given by:

$$E_{g}=2g-r-b$$

$$D_{gr}=\left(g-r\right)/\left(g+r\right)$$

$$D_{gb}=\left(g-b\right)/\left(g+b\right)$$

where *g*, *r* and *b* are green, red and blue component values, respectively.
(4) Classification Learner in MATLAB R2016a was used to select appropriate indices based on color feature analysis as feature values for classification. Two classifiers, support vector machine (SVM) and K nearest neighbor (KNN), were trained (Table 1). The one with the better accuracy based on the training results was chosen as the classier.
(5) The selected classifier was then applied to superpixel classification for preprocessed images. The classification results were subjected to simple morphological processing (removing the fragments) to obtain the final wheat spike, namely wheat spikes recognition.

**TABLE 1:** Different types of classifier features.

Classifier type	Prediction speed	Memory usage	Interpretability	Model flexibility
linSVM	Binary: Fast Multiclass: Medium	Medium	Easy	Low Makes a simple linear separation between classes
quaSVM	Binary: Fast Multiclass: Slow	Binary: Medium Multiclass: Large	Hard	Medium
cubSVM	Binary: Fast Multiclass: Slow	Binary: Medium Multiclass: Large	Hard	Medium
finGSVM	Binary: Fast Multiclass: Slow	Binary: Medium Multiclass: Large	Hard	High, creases with kernel scale setting Makes finely detailed distinctions between classes, with kernel scale set to sqrt(P)/4
medGSVM	Binary: Fast Multiclass: Slow	Binary: Medium Multiclass: Large	Hard	Medium Medium distinctions, with kernel scale set to sqrt(P)
coaGSVM	Binary: Fast Multiclass: Slow	Binary: Medium Multiclass: Large	Hard	Low Makes coarse distinctions between classes, with kernel scale set to sqrt(P)*4, where P is the number of predictors
finKNN	Medium	Medium	Hard	Finely detailed distinctions between classes. The number of neighbors is set to 1
medKNN	Medium	Medium	Hard	Medium distinctions between classes. The number of neighbors is set to 10
coaKNN	Medium	Medium	Hard	Coarse distinctions between classes. The number of neighbors is set to 100
cosKNN	Medium	Medium	Hard	Medium distinctions between classes, using a cosine distance metric. The number of neighbors is set to 10
cubKNN	Slow	Medium	Hard	Medium distinctions between classes, using a cubic distance metric. The number of neighbors is set to 10
weiKNN	Medium	Medium	Hard	Medium distinctions between classes, using a distance weight. The number of neighbors is set to 10

### Determination of Wheat Spike Number

**FIGURE 4:**
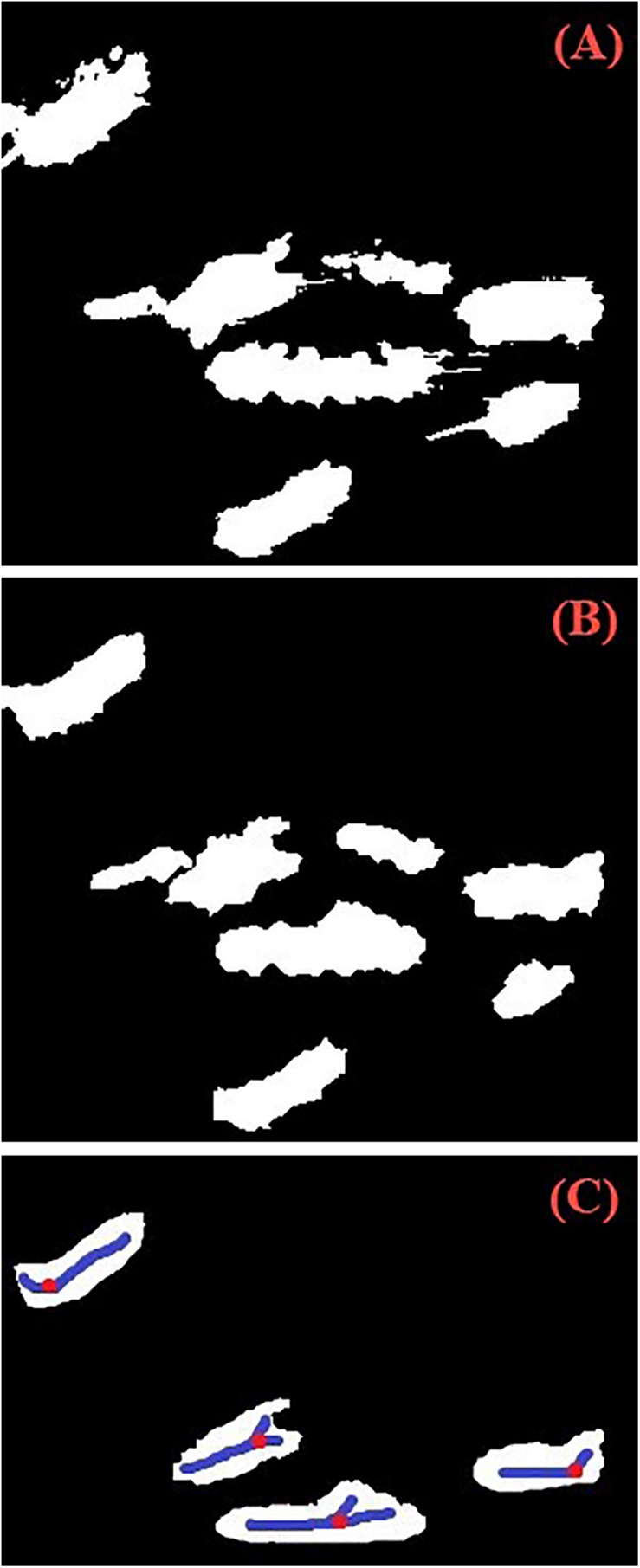
Wheat spikes counting process. [**(A)** Segmentation of wheat spikes: binarization of recognition results. **(B)** Morphological transformation: mathematical morphology open operator was used to reduce the disturbances. **(C)** Skeleton inflection point in the overlap area of wheat spikes: the overlapping region was detected according to the shape and area parameters of the region].

As shown in Figure 4, the wheat spike recognition results were binarized. During wheat spike counting, greater emphasis was given to the main part of spikes and not to morphology. The main part of spikes was preserved by morphological transformation such as erosion and dilation operation (Possa et al., 2014), while the edges were weakened to reduce adhesion to adjacent spikes. Statistics were performed for the regions in the binarized images. Traits such as number (*n*_*region*_) and area of regions in the binarized images were calculated. In addition to the complex field environment, the grains were plump and the spikes were larger in size during the grain filling stage. Therefore, the binarized images after morphological transformation still contained a few wheat spike overlap. These overlaps were screened based on the area and morphology (length-to-width ratio) of the regions. Backbone of the head was extracted from the overlaps (Cremers et al., 2007; Delgado-Friedrichs et al., 2015) and the number of inflection points of backbone (Liu et al., 2001) (*n*_*point*_) was calculated. The wheat spike number for the overlaps was *n_*point*_* + 1, and the total wheat spike number was *n*_*region*_ + *n*_*point*_.

### Statistical Analysis

SPSS 22.0 software was used for statistical analysis. The relationship between automatic count and artificial count was analyzed based on Pearson’s correlation coefficient (r) and linear regression analysis. Such correlations were compared under different nitrogen fertilizer application levels, and the accuracy (*A*) was calculated by using the artificial count as benchmark. Thus accuracy of wheat spike number estimate was compared under different nitrogen fertilizer application levels to discuss the influence of growth status on the recognition effect. Accuracy *A* was given below:

$$A=\left(1-\frac{\left|N_{c}-N_{a}\right|}{N_{a}}\right)\times 100\%$$

where *N*_*c*_ is the automatic count; *N*_*a*_ is the artificial count; *A* is accuracy.

## Results and Analysis

### Wheat Spike Recognition

#### Analysis of Classification Features

**FIGURE 5:**
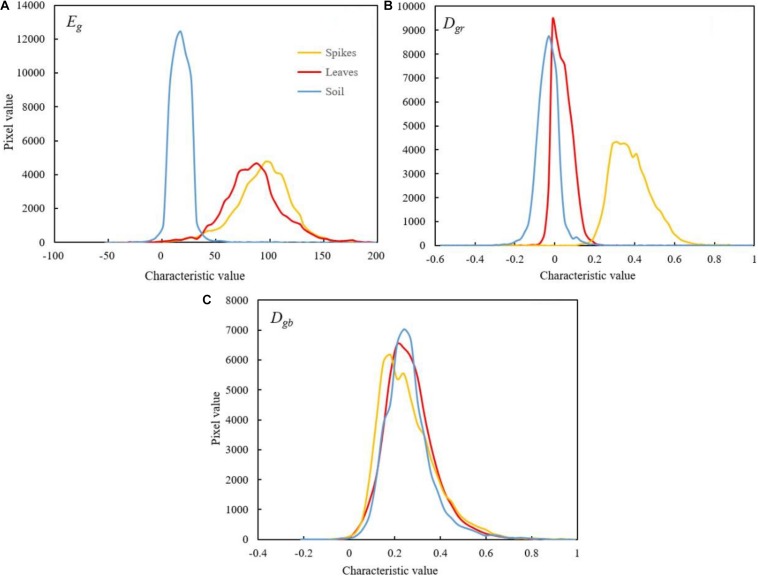
Color histogram of wheat spike, leaf and soil samples. [**(A)** Super green index, E_*g=*_2*g*-*r*-*b*. **(B)** Normalized red green index, D_*gr=*_ (*g*-*r*)/(*g*+*r*). **(C)** Normalized blue green index, D_*gb*_ = (*g*-*b*)/(*g*+*b*). The yellow, red and blue lines represented spikes, leaves and soil, respectively].

Three color indices associated with G component, namely, super green value (*E*_*g*_), normalized red green index (*D*_*gr*_) and normalized blue green index (*D*_*gb*_), were applied to the calculation in wheat spike, leaf and soil samples during the grain filling stage, respectively, (Figure 5). The results showed that *E*_*g*_ of soil samples was generally smaller and had a concentrated distribution, with nearly no overlap with the spikes; the distribution range and curve morphology of *E*_*g*_ in leaf and wheat spike samples were very close to each other, resulting in severe overlap. Therefore, *E*_*g*_ was fit for differentiation between spikes and soil, but not for reducing leaf interferences in the images (Figure 5A). *D*_*gr*_ of the soil samples was generally small and there was serious overlap in the above-zero part with the curve of wheat spikes. However, *D*_*gr*_ of leaf samples was distributed within a broader range, showing little overlap with the curve of wheat spike samples and having large peak difference, which was helpful to discriminate between the wheat spikes and leaves. *D*_*gb*_ curves of the three types of samples almost coincided with each other, indicating little value for wheat spike recognition.

#### Results of Classifier Training

*E*_*g*_ and *D*_*gr*_ were chosen as classification features to train the SVM and KNN classifiers (Table 1) in the MTLAB R2016a toolbox. Then the appropriate classifier was chosen based on the training results (Table 2). The results showed that the classifier accuracy was 80% without nitrogen fertilizer application (N1), and *medGSVM* took on the highest level with the accuracy of 85.63%. Under low nitrogen fertilizer application level (N2), different classifiers varied little in accuracy, which was generally around 88%. *finGSVM* was the optimal classifier under this level, with accuracy reaching 90.93%. Under normal nitrogen fertilizer application level (N3), nearly all classifiers had accuracy above 90%. *medGSVM* was the optimal one, with the accuracy of 93.82%. Under high nitrogen fertilizer application level (N4), all classifiers had accuracy above 90%. *cubSVM* was the optimal one under this level, with the accuracy of 94.01%. Under mixed nitrogen fertilizer application level, *weiKNN* was the optimal classifier with the accuracy of 90.61%. In a word, SVM classifiers had a higher performance under single nitrogen fertilizer application level and had higher accuracy; but under mixed nitrogen fertilizer application level, KNN classifiers outperformed SVM classifiers in terms of accuracy. As the nitrogen fertilizer application level increased, the classification accuracy also rose and trended to a stable level. For example, as compared with N1 level, the classifiers had an improvement of accuracy by 5.3% under N2 level; as compared with N2 level, the accuracy increased by 2.89% under N3 level; the accuracy under N4 level only improved by 0.19% as compared with N3 level. On the whole, the classification accuracy was close with the two classifiers on samples with nitrogen fertilizer application (N2, N3 and N4). The growth status of wheat seedlings was worse without nitrogen fertilizer application, and there were considerable differences in uniformity, color and size as compared with those with nitrogen fertilizer application. Therefore, the greater the heterogeneity within the mixed samples, the lower the classification accuracy.

**TABLE 2:** Classifier training results (Classification classification accuracy,%; single nitrogen level, *n* = 3 000; mixed nitrogen level, *n* = 10 000).

Classifier type	Nitrogen level				
	N1	N2	N3	N4	Mix
linSVM	80.54	87.65	90.48	90.26	83.57
quaSVM	84.55	88.09	91.25	91.13	87.02
cubSVM	85.04	87.89	91.54	94.01	55.78
finGSVM	83.37	90.93	90.07	92.42	88.61
medGSVM	85.63	88.87	93.82	91.03	86.61
coaGSVM	80.92	87.43	90.97	90.17	84.75
finKNN	83.47	87.89	87.43	90.64	83.67
medKNN	84.26	88.28	90.64	91.33	83.67
coaKNN	81.90	86.81	89.17	90.26	85.63
cosKNN	83.27	87.79	90.46	90.33	85.53
cubKNN	83.96	88.18	89.34	90.75	85.53
weiKNN	84.84	89.46	90.48	91.54	90.61

*Single nitrogen level (N1, N2, N3, N4), *n* = 3 000; Mixed nitrogen level, *n* = 10 000.*

#### Results of Wheat Spike Recognition

**FIGURE 6:**
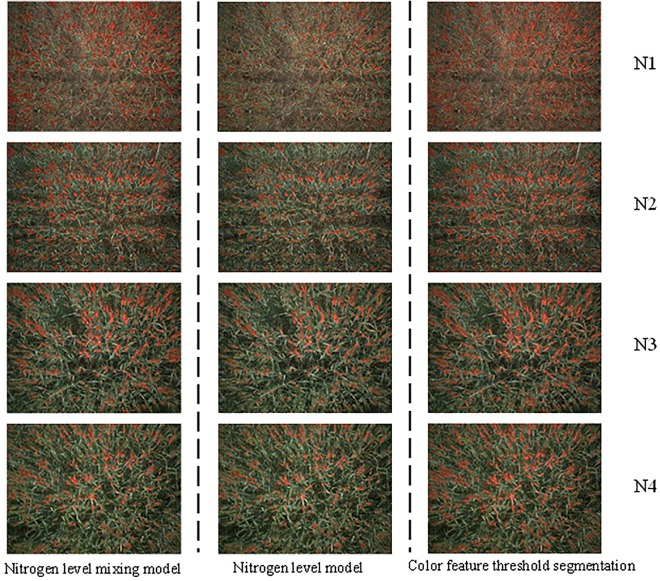
Wheat spikes recognition results. [For nitrogen level mixing model, nitrogen level model, and color feature threshold segmentation model, recognition under different nitrogen level gradient. The left picture was nitrogen level mixing model, the middle picture was nitrogen level model, the right picture was color feature threshold segmentation. N1, N2, N3 and N4 represented 0 (nitrogen-free), 225 kg⋅ha^–1^ (1/2 of the normal nitrogen level), 450 kg⋅ha^–1^ (normal nitrogen level), and 900 kg⋅ha^–1^ (excessive nitrogen level), respectively].

Based on the above analysis, the classifiers established were applied to wheat spike classification recognition from preprocessed images (Figure 6). As control, color features *E*_*g*_ and *D*_*gr*_ were used for automatic thresholding. The results of two thresholding segmentation were superimposed for pixel-wise segmentation of spikes. The results showed that the recognition effect was better with superpixel classifiers under single nitrogen fertilizer application level than under the mixed level, with significantly less leaf confounding. As compared with pixel-based thresholding segmentation, superpixel recognition led to higher integrity of the main body of spikes, effective improvement of patches in spikes, and better representation of spikes in morphology and size. As the nitrogen fertilizer application level increased, the wheat spike morphology was more clearly visualized and the recognition effect was improved. The spikes in regions with nitrogen fertilizer application (N2, N3 and N4) could be all effectively recognized. The actual classification results were consistent with the training results of the classifiers.

### Wheat Spikes Number Estimate

#### Wheat Spike Number Estimate Under Different Nitrogen Fertilizer Application Levels

**FIGURE 7:**
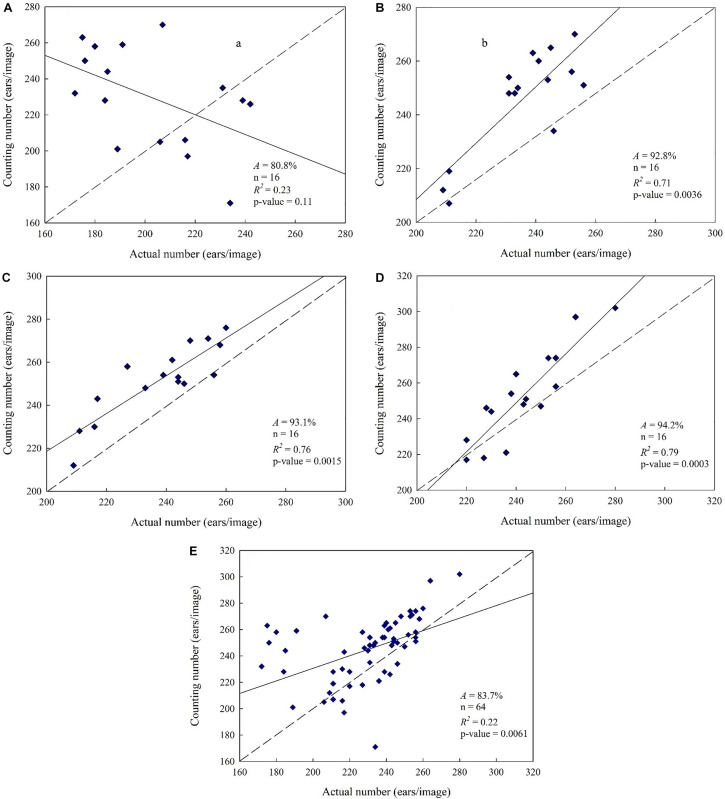
Wheat spikes recognition results. [**(A)** Nitrogen-free, 0 kg⋅ha^–1^. *A* = 80.8%, *n* = 16, *R*^2^ = 0.23, *p*-value = 0.1. **(B)** Low-nitrogen, 225 kg⋅ha^–1^. *A* = 92.8%, *n* = 16, *R*^2^ = 0.71, *p*-value = 0.0036. **(C)** Normal nitrogen, 450 kg⋅ha^–1^. *A* = 93.1%, *n* = 16, *R*^2^ = 0.76, *p*-value = 0.0015. **(D)** High nitrogen, 900 kg⋅ha^–1^. *A* = 94.2%, *n* = 16, *R*^2^ = 0.79, *p*-value = 0.0003. **(E)** Mixed nitrogen, samples were mixed at four nitrogen levels. *A* = 83.7%, *n* = 64, *R*^2^ = 0.22, *p*-value = 0.0061].

The samples were mixed together under single nitrogen fertilizer application level and under mixed level, respectively. The classifiers were trained for wheat spike extraction based on superpixel blocks. Then based on wheat spike recognition result, the wheat spike number was automatically determined. The artificially counted wheat spike number was used as benchmark to calculate the accuracy. Linear regression was performed between automatic count and actual count to form a 1:1 relationship diagram (Figure 7). Automatic counts were compared under different nitrogen fertilizer application levels. The overall accuracy was 80.8% under no nitrogen fertilizer application. Severe deviation was observed in the automatic count under a small wheat spike number, and the correlation between automatic count and artificial count was also worse (*R^2^* = 0.23, *p* > 0.05), respectively. That is to say, the difference was of no statistical significance and the automatic count was less satisfying. By contrast, higher accuracy was achieved under all other three nitrogen fertilizer application levels (*A*_*low*_ = 92.8%, *A_*normal*_* = 93.1%, *A_*high*_* = 94.2%). Besides, there was good correlation between automatic count and actual count (*R^2^_*low*_* = 0.71, *R^2^_*normal*_* = 0.76, *R^2^_*high*_* = 0.79), which was of extreme statistical significance (*P* < 0.01). The accuracy of automatic wheat spike number estimate was generally high. As the nitrogen fertilizer application level increased, the automatic wheat spike number estimate was also improved and it was the best under high nitrogen fertilizer application level, with accuracy reaching up to 94.2%. Automatic wheat spike number estimate was based on processing and statistics of regions in binarized image of wheat spike segmentation. Therefore, the automatic wheat spike number estimate and wheat spike segmentation were consistent under different nitrogen fertilizer application levels. However, the classifiers were of poor applicability under no nitrogen fertilizer application, while all statistics were effective for regions with nitrogen fertilizer application. When all samples were mixed together regardless of the nitrogen fertilizer application level, the accuracy of automatic wheat spike number estimate was 83.7%, and *R*^2^ was 0.22. Under the condition of 240 spikes per image in the present study, this accuracy was quite low and could not meet the requirements of wheat production practice.

### Wheat Spike Number Estimates for Plots Applied With Nitrogen Fertilizer

**FIGURE 8:**
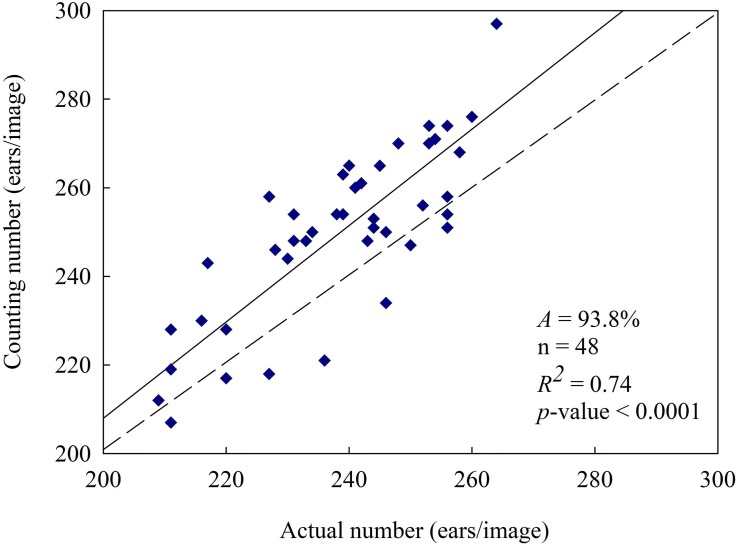
Wheat spikes counting result in nitrogen application. [Eliminate 16 nitrogen-free samples and mix the remaining 48 samples for identification verification. The result was *A* = 93.8% and *R*^2^ = 0.74, at *p*-value < 0.0001 level].

Based on the above results, samples under no nitrogen fertilizer application were removed from the training samples. In order to establish representative mixed samples set applied to train the classifiers, the remaining samples were mixed and used to train the classifiers after K-means clustering. Overall accuracy and wheat spike segmentation accuracy was determined according to the training results (Table 3), and *weiKNN* classifier was further used for superpixel classification to extract the spikes. Finally, automatic wheat spike number estimate was obtained (Figure 8). As compared with the statistics under the mixed nitrogen fertilizer application level (Figure 7E), the estimate performance was significantly improved. Accuracy increased from 83.7 to 93.8%, and the correlation between the automatic count and actual count was also improved significantly, with *R*^2^ rising from 0.22 to 0.74. Therefore, in regions with nitrogen fertilizer application, segmentation and statistics might be performed without considering the differences in nitrogen level, and the estimate is reliable.

**TABLE 3:** Classification training results of nitrogen application samples (*n* = 10 000).

Classifier type	Overall accuracy (%)	Wheat spike accuracy (%)	Background accuracy (%)
linSVM	86.40	87.30	85.31
quaSVM	88.49	90.27	86.30
cubSVM	83.33	76.38	90.27
finGSVM	88.49	88.29	89.28
medGSVM	89.78	91.27	88.30
coaGSVM	87.30	89.28	85.31
finKNN	86.70	86.30	86.30
medKNN	88.68	89.28	88.29
coaKNN	88.39	92.26	85.31
cosKNN	88.09	87.30	89.28
cubKNN	88.19	88.29	87.30
weiKNN	91.48	93.81	89.30

## Discussion

The accuracy of automatic ear number estimate relies upon reliable segmentation of ear images. Among the existing image segmentation methods, image features extracted based on target features mainly included color, texture and shape (Caelli and Reye, 1993; Mukherjee et al., 2015). However, texture features received large interference from the leaves in the process of spike image segmentation during the grain filling stage, and the segmentation effect was less satisfactory (Felzenszwalb and Huttenlocher, 2004). Moreover, due to large number of wheat plants during the grain filling stage, the spikes overlapped with each other, which increased the difficulty in extracting shape features as well. According to field observation, wheat spikes changed substantially in color during the grain filling stage, namely, from green to yellowish green, while the stalks and leaves still remain green. So, color features were fit for wheat spike image segmentation at this stage, which was in agreement with previous researches. Besides, most of the image segmentation algorithms were based on pixels while ignoring the inherent spatial relationship between the pixels. As a result, the image processing effect was poor under non-structured natural scenes (Zhang et al., 2017). Recent years have witnessed an increasing application of remote sensing technology. For large-scale remote sensing images, pixel-based segmentation could hardly meet the requirements on calculation efficiency. Compared with pixels, superpixels had the following advantages: effective utilization of spatial relationship between pixels, reducing object scale and complexity of subsequent processing, while increasing processing efficiency (Alex et al., 2009; Derksen et al., 2019). Based on previous studies (Mangasarian and Wild, 2006; Xiong et al., 2017), we used superpixel segmentation for image pre-processing and extracted color information as wheat spike features, so as to improve the wheat spike recognition effect.

Then, based on the extracted image features, SVM and KNN classifiers were applied to wheat spike recognition, respectively. The results showed that the SVM classifier outperformed KNN classifier under a single nitrogen fertilizer application level and had higher classification accuracy. But for mixed samples, the accuracy of KNN classifier was higher than that of SVM classifier. SVM, built upon the theory of statistical learning, was more adapted to the classification problems with small sample size, non-linearity and high dimensionality. However, the recognition effect might be poor when applied to the classification problems with large number of training samples and support vectors (Weinberger et al., 2009). KNN, a classical lazy learning algorithm, usually has the features of large computation load and low efficiency, but for samples with much overlap or of large scale, it might be a favored method (Zhang and Zhou, 2007). In this study, SVM classifier outperformed the KNN classifier when the sample size was small, while KNN classifier was better in the case of larger sample size, which is typical of the two types of classifiers.

Moreover, recognition results under different nitrogen fertilizer application levels indicated a worse wheat spike recognition effect without nitrogen fertilizer application than with nitrogen fertilizer application. Wheat seedlings grown on nitrogen-deficient soils would have tender stalks and yellowish green leaves, stalks and spikes. In that case, the use of color features extracted from the images for wheat spike recognition would lead to misclassification and poor recognition effect. Moreover, Nitrogen status affects the accumulation of dry matter and nitrogen in the spike (Demotes-Mainard and Jeuffroy, 2004). Wheat spikes are short and small when nitrogen is deficient. They might be easily misinterpreted as background fragments and removed in post-classification processing, leading to severe missed classification. That is why the wheat spike recognition effect is poor without nitrogen fertilizer application.

Here, digital images of wheat in field environment during the grain filling stage were subject to classification. After flowering, the grains were fertilized and the spikes kept expanding in size. During the grain filling stage, the spikes would finally grow to its maximum size, and spikes would be the main components in the digital images at this stage. But in the flowering stage, leaves took up higher proportion in the images, while the proportion of spikes were lower, which was not conducive to feature extraction and analysis. During the maturity stage after the grain fillings stage, as the grains mature and leaves age, the wheat seedlings on the whole showed a golden color, with little distinction between the spikes and stalks. Moreover, due to disturbance from the soil background in the images, it was difficult to extract image features. Therefore, we believed that the grain filling stage was the optimal time for wheat spike recognition. Given the distinct morphology and color changes of wheat during the grain filling stage, we only chose digital images of wheat during this single stage. Field image analysis and wheat spike recognition of wheat during multiple reproductive stages were worthy of further investigation. Field environment might be complex for wheat spike image acquisition due to a diversity of leave and wheat spike postures, which further leaded to variation of illumination conditions even for the same spikes. As the color features of spikes in the images vary significantly, the accuracy of image segmentation based on color features would be impaired. All of the above factors could affect the wheat spike recognition effect, and more studies should be conducted to find out an appropriate method for image acquisition and processing which better represents wheat spike feature. The methods are important for improving the wheat spike recognition effect and accuracy of wheat spike number estimate.

Furthermore, through color histogram analysis, wheat spikes during the grain filling stage were effectively recognized based on color features *E*_*g*_ and *D*_*gr*_. As compared with pixel-based segmentation, segmentation based on superpixel block produced more intact wheat spike morphology, better preserved edge information and reduced missed classification. The growth status of wheat seedlings varied under different nitrogen fertilizer application levels. The best wheat spike recognition effect was achieved under higher nitrogen fertilizer application level (*A*_*high*_ = 94.2%), and the effect was also good under normal (*A*_*normal*_ = 93.1%) and low (*A*_*low*_ = 92.8%) nitrogen fertilizer application levels. The recognition effect was the worst without nitrogen fertilizer application (*A_*no nitrogen*_* = 80.8%). For mixed samples, after excluding those under no nitrogen fertilizer application, the wheat spike number estimate was improved significantly, with accuracy reaching up to 93.8%, which was a 10.1% increase. To conclude, automatic wheat spike number estimate based on superpixel segmentation and color features is a rapid and accurate method that applies to the general field environment. However, this method is not recommended for use when the growth status of wheat is poor or when the regions are of high heterogeneity.

Deep learning methods have been currently very popular (Sadeghi-Tehran et al., 2019), but their methods are mainly based on a large amount of sample data and high-configuration hardware. Sample preparation requires a lot of manpower and time. Compared with the complex neural network algorithms, this method based on traditional machine vision superpixel segmentation is more easily accepted. It is not limited by the performance of hardware computing, simple and efficient, and has certain stability. This experimental method has high accuracy in wheat spikes recognition and is suitable for popularization and application.

## Data Availability Statement

All datasets generated for this study are included in the article/supplementary material.

## Author Contributions

CT and WG conceived the research. CT, PZ, and XZ designed and performed the experiments. CT, YZ, YD, and ZW prepared and revised the manuscript. CT and DW analyzed the data. WM and WL provided technical. All authors discussed the results and commented on the manuscript.

## Conflict of Interest

The authors declare that the research was conducted in the absence of any commercial or financial relationships that could be construed as a potential conflict of interest.
